# P-1250. Pharmacokinetic Evaluation of Colistin Methanesulfonate in Critically Ill Patients: Implications for Clinical Management

**DOI:** 10.1093/ofid/ofae631.1432

**Published:** 2025-01-29

**Authors:** Riya Mary Jose

**Affiliations:** Mary Queen's Mission Hospital, Kanjiramattam, Kerala, India

## Abstract

**Background:**

The study aims to assess the pharmacokinetics of colistin methanesulfonate (CMS) and colistin, and its impact on clinical outcomes, particularly clinical cure (CC), and the development of acute kidney injury (AKI) in critically ill patients with normal baseline renal function.

**Methods:**

This prospective observational study focuses on adult critically ill patients with MDR/XDR infections treated with intravenous CMS. Patients receive a loading dose (LD) of 9 MU followed by a maintenance dose (MD) of 3 MU every 8 hours. Pharmacokinetic (PK) sampling occurs pre- and post-LD, and during MD. Colistin plasma levels are quantified using liquid chromatography–mass spectrometry (LC–MS). Clinical outcomes, including clinical cure, impact on renal failure and mortality, are assessed.

**Results:**

A total of 144 serum samples were analyzed from the 12 patients, with 60% having pneumonia. Predominant pathogens were Klebsiella pneumoniae (7) and Acinetobacter spp. (5). The mean estimated creatinine clearance (CrCl) was 120 ± 22 mL/minute (range: 75.4–215.2). All patients received combination therapy, with 80% receiving meropenem and 40% receiving tigecycline. The clinical cure rate was 50% (6/12), with a mortality rate of 25% (3/12). The mean LD colistin Cmax was 3.1 ± 1.2 mg/L (range: 1.6–5.5) and 2.4 ± 1.3 mg/L (range: 1.4–5.8) among the CC and CF groups, respectively (P = 0.14). MD colistin Css avg was 2.3 ± 1.4 mg/L and 1.8 ± 1.2 mg/L in CC and CF groups, respectively. The mean AUC0–24/MIC ratio of MD colistin was 94.3 ± 68.2 and 78.1 ± 53.6 for CC and CF groups, respectively (P = 0.28). In pneumonia, AUC0–24/MIC for Acinetobacter spp. was higher in the CC (72.1 ± 11.3) than in the CF group (41.5 ± 15.8) (P = 0.06). Renal injury was observed in 40% (5/12) of patients at the end of therapy, with acute kidney injury (AKI) being the most common complication. Additionally, a subset of patients (n = 3) demonstrated evidence of chronic kidney disease (CKD) progression, highlighting the nephrotoxic potential of colistin therapy.

**Conclusion:**

The findings underscore the importance of therapeutic drug monitoring in optimizing colistin therapy and the necessity for vigilant monitoring of renal function to mitigate the risk of AKI and CKD progression.

**Disclosures:**

**All Authors**: No reported disclosures

